# The Longitudinal Association Between Self‐esteem and Depressive Symptoms in Adolescents: Separating Between‐Person Effects from Within‐Person Effects[Fn per2179-note-0064]

**DOI:** 10.1002/per.2179

**Published:** 2018-11-05

**Authors:** M. Masselink, E. Van Roekel, B.L. Hankin, L. Keijsers, G.M.A. Lodder, J. Vanhalst, M. Verhagen, J.F. Young, A.J. Oldehinkel

**Affiliations:** ^1^ Interdisciplinary Center Psychopathology and Emotion Regulation (ICPE) University Medical Center Groningen, University of Groningen The Netherlands; ^2^ Department of Developmental Psychology Tilburg University The Netherlands; ^3^ Department of Psychology University of Illinois at Urbana–Champaign USA; ^4^ Interuniversity Centre for Social Science Theory and Methodology (ICS), Department of Sociology University of Groningen Groningen The Netherlands; ^5^ Department of School Psychology and Development in Context KU Leuven Belgium; ^6^ Behavioural Science Institute Radboud University Nijmegen The Netherlands; ^7^ Department of Child and Adolescent Psychiatry and Behavioral Sciences Children's Hospital of Philadelphia USA

**Keywords:** self‐esteem, depression, random intercept cross‐lagged panel model, within‐person effects, longitudinal data

## Abstract

Many longitudinal studies have investigated whether self‐esteem predicts depressive symptoms (vulnerability model) or the other way around (scar model) in adolescents. The most common method of analysis has been the cross‐lagged panel model (CLPM). The CLPM does not separate between‐person effects from within‐person effects, making it unclear whether the results from previous studies actually reflect the within‐person effects or whether they reflect differences between people. We investigated the associations between self‐esteem and depressive symptoms at the within‐person level, using random intercept cross‐lagged panel models (RI‐CLPMs). To get an impression of the magnitude of possible differences between the RI‐CLPM and the CLPM, we compared the results of both models. We used data from three longitudinal adolescent samples (age range: 7–18 years; study 1: *N* = 1948; study 2: *N* = 1455; study 3: *N* = 316). Intervals between the measurements were 1–1.5 years. Single‐paper meta‐analyses showed support for small within‐person associations from self‐esteem to depressive symptoms, but not the other way around, thus only providing some support for the vulnerability model. The cross‐lagged associations in the aggregated RI‐CLPM and CLPM showed similar effect sizes. Overall, our results show that over 1‐ to 1.5‐year time intervals, low self‐esteem may negatively influence depressive symptoms over time within adolescents, but only weakly so. © 2018 The Authors. European Journal of Personality published by John Wiley & Sons Ltd on behalf of European Association of Personality Psychology

Adolescence is a period marked by many social, physical, and emotional changes, and going through these transitions has its effects on adolescents' self‐esteem and depressive symptoms (Hankin, 2006). Many studies have shown that self‐esteem and depressive symptoms are associated with each other in adolescents, with lower self‐esteem being associated with more depressive symptoms (Masselink, van Roekel, & Oldehinkel, 2017; Sowislo & Orth, 2013). Understanding the nature of this association can have great theoretical and clinical benefits. Beck's cognitive theory of depression (Beck, 1967) and hopelessness theory (Abramson & Metalsky, 1989; Metalsky, Joiner, Hardin, & Abramson, 1993) conceptualize low self‐esteem as an important vulnerability factor for the development of depression. In contrast to the vulnerability model, the order of effects is reversed in the scar model (Lewinsohn, Steinmetz, Larson, & Franklin, 1981; Watson & Clark, 1995). The scar model assumes that going through a depression can have long‐lasting negative effects on personality and self‐concept (e.g. self‐esteem), even after recovering from the depression (Rohde, Lewinsohn, & Seeley, 1990; Shahar & Davidson, 2003). The depressive episode is thus assumed to leave a psychological ‘scar’ on one's self‐esteem. Scar models are difficult to test because they require premorbid and postmorbid diagnostic measures (e.g. Ormel, Oldehinkel, & Vollebergh, 2004). In research investigating the association between self‐esteem and depression, depression is usually measured on a continuous scale and a scar effect is often operationalized as a negative longitudinal effect of depressive symptoms on self‐esteem, without taking into consideration diagnostic status or whether depressive symptoms are still present on the last measurement moment (e.g. Orth et al., 2008; Sowislo & Orth, 2013). We will follow this simplified operationalization of a scar effect. Overall, the vulnerability model predicts that low self‐esteem leads to depression, and the scar model predicts that depression leads to low self‐esteem. Of course, these models are not mutually exclusive; reciprocal associations between self‐esteem and depression are possible.

Over the past 30 years, the abovementioned models have been tested in several longitudinal studies. A major advantage of longitudinal studies is that they allow investigation of temporal associations by evaluating the direction of associations between self‐esteem and depressive symptoms over time. Overall, strong support has been found for the vulnerability model, such that low self‐esteem seems to make adolescents vulnerable to developing depressive symptoms (Sowislo & Orth, 2013). This effect has been shown to be very robust, to occur in boys as well as girls, and to remain present after controlling for content overlap between self‐esteem and depression and for Big Five personality traits (Masselink et al., 2017; Sowislo, Orth, & Meier, 2014). Support has been found for the scar model as well (e.g. Schiller, Hammen, & Shahar, 2016; Shahar & Davidson, 2003), but scar effects have been found less often than vulnerability effects and seem to be smaller (about half the effect size; Sowislo & Orth, 2013).

Taken together, it seems as if the existing research has provided solid evidence for longitudinal associations between low self‐esteem and depressive symptoms in adolescents. Yet we argue that this assumed basic understanding about the associations between self‐esteem and depressive symptoms may not be as solid as presumed. The reason for this is that there are significant limitations in the research methods that have been used.

The vulnerability and scar models both describe processes that occur *within persons*. That is, an individual's own self‐esteem is hypothesized to relate to that same individual's risk of becoming depressed and vice versa. Thus, during or following periods in which a person has lower levels of self‐esteem, this person is at increased risk for depression (the vulnerability model), or during or following periods in which a person has increased depressive feelings, this person's self‐esteem may go down (scar model). In contrast, a *between‐person* hypothesis would be that adolescents who have lower self‐esteem compared with other adolescents are more likely to be depressed compared with other adolescents (and vice versa). When the goal is to empirically test theories that describe within‐person associations, which is the case for the vulnerability and scar models, the method of analysis should be in line with this goal. Longitudinal studies examining the association between self‐esteem and depressive symptoms typically used the cross‐lagged panel model (CLPM) (e.g. Orth, Robins, Meier, & Conger, 2016; Orth, Robins, Widaman, & Conger, 2014; Rieger, Göllner, Trautwein, & Roberts, 2016). A CLPM consists of (i) paths between different constructs measured at the same time point (e.g. the cross‐sectional association between self‐esteem and depression at T1), (ii) rank‐order stability paths, also known as auto‐regressive paths, which are paths over time between the same constructs (e.g. the association between depression at T1 and T2), and (iii) cross‐lagged paths, which are paths over time between the different constructs (e.g. the association between depression at T1 and self‐esteem at T2), after controlling for the auto‐regressive effect.

A major limitation of the CLPM is that it does not separate between‐person effects from within‐person effects. Depending on the between‐person and within‐person variance structures, CLPM results may reflect mostly between‐person effects, mostly within‐person effects, or an ambiguous mix of effects, leaving the researcher with an uninterpretable blend of effects (Berry & Willoughby, 2016). Recently, it has been argued that effects found between persons only generalize to the individual under very strict assumptions, which are hardly ever met in real data (Berry & Willoughby, 2016; Curran & Bauer, 2011; Hamaker, 2012; Molenaar & Campbell, 2009). Using simulated data, for instance, Hamaker, Kuiper, and Grasman (2015) showed that when constructs are to some extent trait like, results from the CLPM may result in erroneous conclusions about within‐person relationships with regard to presence, direction, and the strength of associations. Two recent applied studies found that CLPM results indeed differed from the within‐person associations (Keijsers, 2016), with even opposite within‐person effects (Dietvorst, Hillegers, Hiemstra, & Keijsers, 2017). Although self‐esteem and depressive symptoms are not entirely stable over time, both have clear trait components (Robins & Trzesniewski, 2005) and may be at risk of being wrongfully interpreted as within‐person associations in a CLPM. Theoretically, it is unlikely that the association between self‐esteem and depression is reversed within persons, thus that adolescents would be at increased risk for developing depressive symptoms in or following periods with increased self‐esteem. It is however conceivable that the strength or even presence of the vulnerability effects and scar effects may not be found within persons and the predominance of the vulnerability effect over the scar effect that is found in CLPMs may be absent or reversed within persons. Between‐person confounders may cause lagged effects in a CLPM (for instance, when girls score higher than boys on both depression and self‐esteem problems, a significant lagged relationship may emerge). Removing these confounders, it is possible that within persons, no effects are found or different effects are found (e.g. Dietvorst et al., 2017; Hamaker et al., 2015). For example, within persons, the scar effect may be stronger than the vulnerability effect, or the effect size of the vulnerability effect may be much larger or smaller within persons than what has been found in studies using CLPMs. Given the trait aspects of self‐esteem and depressive symptoms, there is thus a need for studies explicitly focusing on within‐person longitudinal associations between self‐esteem and depressive symptoms. Moreover, within‐person associations may help to identify modifiable targets for intervention (between‐person associations are helpful in detecting who needs an intervention).

To investigate within‐person associations, the CLPM can easily be extended to a random intercept cross‐lagged panel model (RI‐CLPM; Hamaker et al., 2015). 1Although a similar model had already been used before (trait and state model; Ormel, Rijsdijk, Sullivan, van Sonderen, & Kempen, 2002; Spinhoven, Penelo, De Rooij, Penninx, & Ormel, 2014), the RI‐CLPM has recently been named as such and extensively described in terms of between‐person and within‐person effects by Hamaker et al. (2015). An RI‐CLPM controls for all stable unmeasured covariates that affect both self‐esteem and depression (for instance, reporter biases, gender, and socio‐economic status) by taking out variance that is due to stable differences between persons. The RI‐CLPM (see Figure 1 for a schematic representation) splits variance of each variable into a stable time‐invariant trait‐like part (captured with random intercepts) and within‐person fluctuations from measurement to measurement around the person's own expected score (captured with a novel latent factor per measurement wave). The expected score indicates how an individual is expected to score on a given time point based on the sample mean level across time and the individual's stable trait factor. The random intercepts thus indicate the trait‐like between‐person variability across all measures, while the latent factors per measurement indicate the within‐person fluctuations on each time point (for a technical description, see Hamaker et al., 2015, p. 104–105). Let us say, for example, that an individual's scores across all three time points are on average 2 points higher than the population average. This average deviation is used to calculate the expected score for this individual at each time point. If the population average is 1 at T1, then the expected score for this individual is 1 + 2 = 3 at T1. If the individual's actual score is 2.5 at T1, the deviation of the expected score is −0.5. This deviation is captured by the within‐person latent factor on each time point and used to estimate the within‐person associations between constructs and time points. Interpretation of the coefficients in the RI‐CLPM does not relate to stability in rank order and change in rank order, as in the CLPM, but to within‐person changes. The T1 correlation reflects the correlation between deviations from the individual's own expected self‐esteem and depressive symptoms scores. Associations between self‐esteem and depressive symptoms at T2 and T3 reflect whether within‐person changes in self‐esteem are associated with within‐person changes in depressive symptoms. The stability effects reflect carry‐over effects, thus whether deviations from one's own expected self‐esteem or depressive symptoms score at one moment carry over to the next measurement moment. Cross‐lagged effects reflect spillover effects and are to be interpreted as the extent to which individuals' changes in deviation from the expected depressive symptoms score are predicted by deviations from their expected self‐esteem score on the previous measurement moment, after controlling for the carry‐over stability effects, and vice versa.

**Figure 1:**
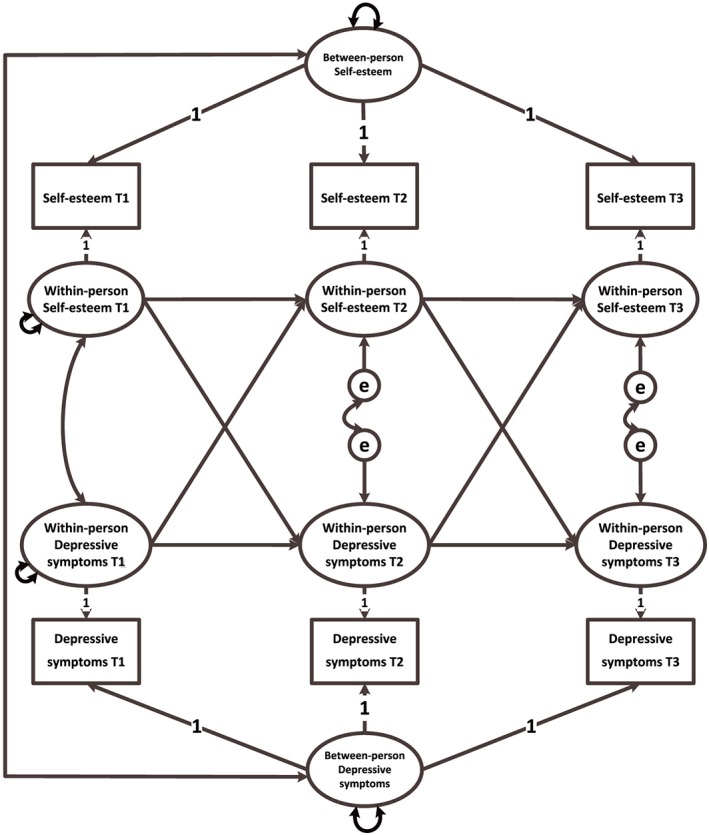
The random intercept cross‐lagged panel model. [Colour figure can be viewed at wileyonlinelibrary.com]

## Present Study

Given the lack of studies examining the longitudinal associations between self‐esteem and depressive symptoms within persons, and the risk of obtaining biased estimates with a CLPM when the concepts under investigation are to some extent trait like, the goal of the present research was twofold. First, we aimed to investigate within‐person longitudinal associations between self‐esteem and depressive symptoms among adolescents. Second, we aimed to investigate the extent to which conclusions differ depending on whether or not between‐person effects are separated from within‐person effects. If results found in an RI‐CLPM replicate in a CLPM in the same samples, the conclusions based on previous CLPM research may not be too far off from the actual process occurring within persons, which would be reassuring. However, if results do not replicate, conclusions about the nature of the within‐person association between self‐esteem and depression may have to be revised. The research questions were investigated by analysing data from three independent adolescent longitudinal datasets, coming from three different countries, covering the age span of 7–18 years (see Table 1 for descriptive information of the three studies).

**Table 1:** Descriptive information across measurement waves and across studies

	Study 1			Study 2			Study 3		
	T1	T2	T3	T1	T2	T3	T1	T2	T3
*N*	1223	1334	1442	944	1069	942	316	273	244
Mean age (*SD*)	12.81 (0.42)	13.94 (0.47)	15.36 (0.62)	15.79 (1.31)	16.62 (1.22)	17.39 (1.27)	11.52 (2.45)	13.05 (2.45)	15.03 (2.42)
% Boys	50.04	48.65	48.68	33.44	34.90	34.34	45.25	45.05	44.67
Self‐esteem mean (*SD*)	3.77 (1.10)	3.60 (1.21)	3.58 (1.17)	3.06 (0.59)	3.13 (0.58)	3.17 (0.56)	3.29 (0.52)	3.38 (0.53)	3.40 (0.54)
Depressive symptoms mean (*SD*)	0.52 (0.42)	0.52 (0.47)	0.51 (0.45)	0.68 (0.52)	0.67 (0.53)	0.66 (0.53)	0.30 (0.23)	0.24 (0.25)	0.20 (0.21)

## Statistical Analysis Plan for the Three Studies

In order to investigate the within‐person longitudinal association between self‐esteem and depressive symptoms, we conducted an RI‐CLPM in each of the three studies, using three measurement waves in all studies. To get a grasp about the variance that could be explained by stable differences between persons versus the variance explained by over time fluctuations within persons, we calculated the intraclass correlations (ICCs) for self‐esteem and for depressive symptoms. The ICC indicates the proportion of between‐person variance and, conversely, the variance explained by fluctuations within persons (1‐ICC) over the measurement waves of one variable. In each study, we first tested an RI‐CLPM following procedures as described by Hamaker et al. (2015). Each observed self‐esteem and depressive symptoms score was decomposed into a stable between‐person part and a within‐person varying part. In order to capture stable trait‐like differences between persons in self‐esteem and depressive symptoms, two overarching random intercept factors were included, that is, one random intercept for each measure. The two random intercept factors reflect the trait aspects of self‐esteem and depressive symptoms over time. The three observed self‐esteem and depression scores were the indicators of each random intercept, with all factor loadings constrained to 1. The within‐person varying part was captured by regressing each observed score on its own latent factor. The resulting six latent factors (i.e. one for self‐esteem and one for depressive symptoms at each of the three measurement waves) were subsequently used to specify within‐time associations, carry‐over stability paths, and cross‐lagged paths. The error variances of the observed scores were constrained to zero, ensuring that all variation in the observed measures was entirely captured by the within‐person and between‐person latent factor structures. Finally, to investigate whether the effects found in the RI‐CLPMs replicate when between‐person and within‐person effects are not separated, we tested classical CLPMs on each dataset. The models were compared on the cross‐lagged effects because they relate to the vulnerability and scar models. Although we refer to both significance level and effect size, we consider the effect size as most relevant, because significance level does not say anything about the size of the effect and because the RI‐CLPM and CLPM differ in model complexity and thus power to detect effects (see Appendix A of the Supporting Information for an elaborate evaluation). Moreover, differences between effect sizes within and between models were not tested for significance because we were interested in overall patterns across studies, rather than testing specific differences in paths within and between models and studies.

The RI‐CLPMs and CLPMs were tested with Mplus 8.0, using full information maximum likelihood (FIML) that can handle missing data and a robust estimator (MLR) to handle non‐normal distribution of the data. Goodness‐of‐fit indices included the chi‐square, comparative fit index (CFI), root mean square error of approximation (RMSEA), and the standardized root mean square residual (SRMR). As the significance level of the chi‐square is highly dependent on the sample size, model evaluations were based on the CFI, RMSEA, and SRMR. Models with CFI values > 0.90 were considered to have acceptable fit and models with a CFI > 0.95 good fit; RMSEA and SRMR values < 0.08 indicate acceptable fit and <0.05 good fit (Bentler & Bonett, 1980; Hu & Bentler, 1999). As is common in cross‐lagged analyses, we tested whether stability paths and cross‐lagged associations could be constrained to be equal over time in all models (i.e. whether the stability and cross‐lagged effects from T1 to T2 were equal to the same associations from T2 to T3). We compared the model fit of a fully free model with a model with all stability and cross‐lagged associations constrained to be equal over time at once. Following recommendations by Chen (2007) for invariance tests of confirmatory factor models, we also considered invariance over time to be established when ∆CFI < 0.010, ∆RMSEA < 0.015, and ∆SRMR < 0.030. Because stability and cross‐lagged paths in RI‐CLPMs and CLPMs could be constrained to be equal over time in all models, described results refer to models in which the stability and cross‐lagged effects were constrained to be equal over time. All presented results in the figures reflect standardized coefficients; unstandardized coefficients are reported in Table 2. Because equality constraints over time were imposed on the unstandardized coefficients rather than the standardized coefficients, the reported standardized coefficients can still differ over time. Covariance matrices of the three studies are provided in Table S1.

**Table 2:** Unstandardized regression coefficients of studies 1–3

	B [95% CI] (*SE*): S → S	*p*‐value	B [95% CI] (*SE*): D → D	*p*‐value	B [95% CI] (*SE*): S → D	*p*‐value	B [95% CI] (*SE*): D → S	*p*‐value
Study 1								
RI‐CLPM	0.25 [0.14, 0.36] (0.06)	<.001	0.33 [0.21, 0.45] (0.06)	<.001	−0.03 [−0.06, −0.00] (0.01)	.032	−0.22 [−0.47, 0.03] (0.13)	.090
CLPM	0.37 [0.31, 0.42] (0.03)	<.001	0.54 [0.49, 0.59] (0.03)	<.001	−0.04 [−0.05, −0.02] (0.01)	<.001	−0.37 [−0.49, −0.25] (0.06)	<.001
Study 2								
RI‐CLPM	0.32 [0.14, 0.51] (0.09)	.001	0.10 [−0.06, 0.26] (0.08)	.205	−0.10 [−0.24, −0.04] (0.07)	.160	−0.02 [−0.14, 0.11] (0.07)	.822
CLPM	0.66 [0.61, 0.70] (0.02)	<.001	0.43 [0.36, 0.50] (0.04)	<.001	−0.17 [−0.22, −0.12] (0.03)	<.001	−0.07 [−0.13, −0.02] (0.03)	.008
Study 3								
RI‐CLPM	0.12 [−0.09, 0.32] (0.10)	.260	0.10 [−0.12, 0.32] (0.11)	.370	−0.12 [−0.20, −0.04] (0.04)	.005	−0.29 [−0.66, 0.08] (0.19)	.126
CLPM	0.48 [0.37, 0.59] (0.05)	<.001	0.35 [0.22, 0.47] (0.06)	<.001	−0.08 [−0.12, −0.03] (0.02)	.001	−0.14 [−0.36, 0.07] (0.11)	.195

	B [95% CI] (*SE*): D ↔ S T1	*p*‐value	B [95% CI] (*SE*): D ↔ S T2	*p*‐value	B [95% CI] (*SE*): D ↔ S T3	*p*‐value	B [95% CI] (*SE*): S ↔ D†	*p*‐value
Study 1								
RI‐CLPM	−0.07 [−0.11, −0.03] (0.02)	.002	−0.11 [−0.15, −0.08] (0.02)	<.001	−0.08 [−0.11, −0.05] (0.02)	<.001	−0.09 [−0.12, −0.05] (0.02)	<.001
CLPM	−0.15 [−0.18, −0.12] (0.02)	<.001	−0.13 [−0.16, −0.10] (0.02)	<.001	−0.10 [−0.13, −0.07] (0.01)	<.001	n/a	
Study 2								
RI‐CLPM	−0.08 [−0.11, −0.05] (0.02)	<.001	0.06 [−0.08, −0.03] (0.01)	<.001	−0.06 [−0.08, −0.04] (0.01)	<.001	−0.12 [−0.15, −0.09] (0.02)	<.001
CLPM	−0.20 [−0.22, −0.17] (0.01)	<.001	−0.08 [−0.10, −0.07] (0.01)	<.001	−0.08 [−0.10, −0.07] (0.01)	<.001	n/a	
Study 3								
RI‐CLPM	−0.04 [−0.06, −0.02] (0.01)	<.001	−0.06 [−0.08, −0.04] (0.01)	<.001	−0.04 [−0.06, −0.02] (0.01)	<.001	−0.02 [−0.04, −0.01] (0.01)	.006
CLPM	−0.06 [−0.08, −0.05] (0.01)	<.001	−0.06 [−0.08, −0.04] (0.01)	<.001	−0.05 [−0.06, −0.03] (0.01)	<.001	n/a	

*Note*: B, unstandardized regression coefficient; *SE*, standard error; S, self‐esteem; D, depressive symptoms; CI, confidence interval.

† Between‐person trait association between self‐esteem and depressive symptoms.

The analyses for study 3 were pre‐registered (https://osf.io/y3zcn/). The analyses performed deviated from the pre‐registration in a few respects. For transparency, we explain how these changes came about. We initially planned to conduct confirmatory factor analyses on the measures, save the factor scores, and use the factor scores as input in the RI‐CLPM. However, adding one‐level measurement models to the RI‐CLPM resulted in a model in which the within‐person and between‐person variances could no longer be accurately teased apart. We therefore decided against extending the RI‐CLPM with a measurement model but to rely on the common practice of using mean scores instead (in line with the earlier studies using RI‐CLPM, e.g. by Hamaker et al., 2015 and Keijsers, 2016). Initially, factor scores were used in studies 1 and 2 as well, but for the abovementioned reason, we re‐ran the analyses in studies 1 and 2 with mean scores instead of factor scores. Some outcomes differed somewhat depending on whether factor or mean scores were used, which explains the differences in the description of the results of studies 1 and 2 in the present article and the short references to these results in the pre‐registration.

The syntaxes, output, and data of all reported studies are openly available via www.osf.io/p7xcj.

## Study 1

### Methods

#### Sample and procedure

Participants for this study were recruited from seven secondary schools in the Netherlands. Data were collected on three time points (T1–T3), each time point one year apart. The T1 measure took place between January and March 2013, and the T3 measure ended between January and March 2015. Participants at T1 were first grade secondary education students (age range: 10–14 years). Passive consent was obtained from parents and active consent from the adolescents. Of the 1366 adolescents who were contacted to participate in the study, 89 adolescents were absent during data collection, 47 adolescents had no parental consent, and 7 adolescents did not want to participate or had a missing consent form. This resulted in a final sample of 1223 adolescents at T1 (see Table 1 for descriptive information). After the T1 assessment, one school dropped out of the study. Additional participants were recruited in the remaining six schools at T2 and T3. At T2, 70% of adolescents had also participated at T1, and at T3, 56% of adolescents had also participated at T1. The missing data pattern was not completely at random according to Little's missing completely at random (MCAR) test (χ^2^ = 67.98, *df* = 45, *p* = .03). Boys were more likely to drop out of the study over time. In total, 1948 participants did partake in the study on at least one of the measurement waves. Not all students provided data on all measures because of planning issues at some of the schools (four, six, and six cases of incomplete data at T1, T2, and T3, respectively). The FIML estimation was used to handle these missing data patterns. All measures were completed on a computer during regular school hours under supervision of undergraduate students. Ethical approval for the study was obtained from the universities' institutional review board (ECG2012‐2711‐701).

#### Measures

##### Self‐esteem

Self‐esteem was measured at all three time points with the single‐item statement ‘I see myself as somebody with a lot of self‐esteem’, answered on a 5‐point Likert scale from 1 ‘*totally disagree*’ to 5 ‘*totally agree*’. Single‐item self‐esteem measures can be a valid and practical alternative to multiple‐item self‐esteem measures (Robins, Hendin, & Trzesniewski, 2001). A Cronbach's alpha coefficient cannot be calculated for this single‐item measure, but an estimate of reliability was assessed using the Heise procedure (Heise, 1969, equation 9; Robins et al., 2001). In this procedure, the correlation between self‐esteem measured at T1 and T2 is multiplied by the correlation between the self‐esteem at T2 and T3, divided by the correlation between self‐esteem at T1 and T3. This calculation resulted in a reliability estimate of .60.

##### Depressive symptoms

Depressive symptoms were measured with the 11‐item Iowa short form of the Center for Epidemiological Studies Depression Scale (Iowa CES‐D; Kohout, Berkman, Evans, & Cornoni‐Huntley, 1993). The Iowa CES‐D assesses depressive symptoms (e.g. ‘I felt sad’) during the past week. The responses were indicated on a 4‐point scale ranging from 0 ‘*rarely to never, <1 day*’ to 3 ‘*usually or always, 5–7 days*’. The reliability of the CES‐D as indicated by Cronbach's alpha ranged from .81 to .85 across the three time points.

### Results

**Figure 2:**
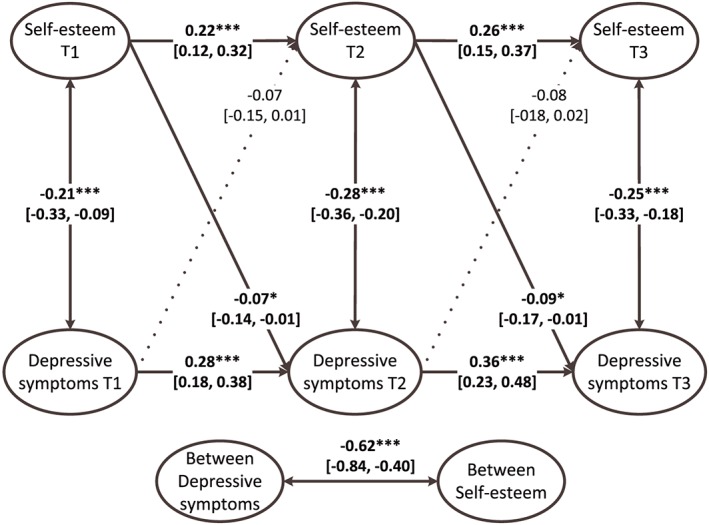
Simplified random intercept cross‐lagged panel model with standardized coefficients from study 1. Numbers between brackets indicate the 95% confidence interval. [Colour figure can be viewed at wileyonlinelibrary.com]

The ICCs for self‐esteem and depressive symptoms indicated that 37% of the variance of self‐esteem could be explained by between‐person differences (63% by fluctuations within persons) and 53% of variance of depressive symptoms could be explained by between‐person differences (47% by fluctuations within persons). The model fit of the constrained RI‐CLPM was excellent, χ^2^(5) = 4.63, *p* = .46, CFI = 1.000, RMSEA = 0.000, SRMR = 0.017 (unconstrained model fit χ^2^(1) = 1.84, *p* = .17, CFI = 0.999, RMSEA = 0.021, SRMR = 0.009). Standardized results are depicted in Figure 2 and unstandardized results in Table 2. The between‐person association between self‐esteem and depressive symptoms was strong and negative, indicating that individuals with higher self‐esteem across the measurement waves reported less depressive symptoms across measurement waves than individuals with low self‐esteem. On the within‐person level, small significant negative concurrent associations were found between self‐esteem and depressive symptoms. Thus, participants who scored higher or lower than their expected self‐esteem score also tended to score higher or lower than their expected depressive symptoms score on T1; and at T2 and T3, participants whose self‐esteem changed from one wave to the other also tended to change in depressive symptoms but in the opposite direction.

The negative within‐person cross‐lagged effects from self‐esteem to depressive symptoms indicated that individuals' deviations from expected depressive symptoms were predicted by their self‐esteem at the previous time point (i.e. individuals who score higher than they typically would on self‐esteem are more likely to score higher on depressive symptoms than they typically would at the next assessment), after controlling for deviations from the expected depressive symptom score on the previous time point. The within‐person cross‐lagged effects from depressive symptoms to self‐esteem were not significant but of almost equal effect size as the effects from self‐esteem to depressive symptoms (*β* = −.07 from T1 to T2 and *β* = −.08 from T2 to T3). There were small positive carry‐over stability paths of self‐esteem and depressive symptoms as well. Within‐person deviations from the expected self‐esteem and depressive symptoms scores thus predicted deviations from the expected self‐esteem and depressive symptoms scores at the next time point.

**Figure 3:**
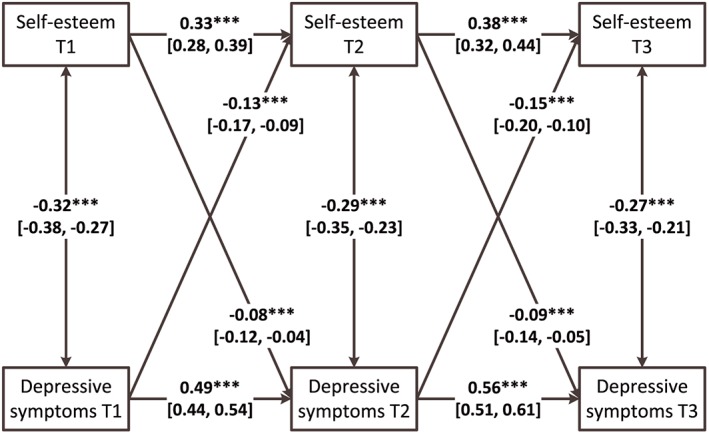
Cross‐lagged panel model with standardized coefficients from study 1. Numbers between brackets indicate the 95% confidence interval. [Colour figure can be viewed at wileyonlinelibrary.com]

As a comparison, the classic constrained CLPM model had a good fit to the data, χ^2^(8) = 30.18, *p* < .001, CFI = 0.978, RMSEA = 0.038, SRMR = 0.037 (unconstrained model fit χ^2^(4) = 30.57, *p* < .001, CFI = 0.973, RMSEA = 0.058, SRMR = 0.035). Standardized results are depicted in Figure 3 and unstandardized results in Table 2. There were significant small to moderate concurrent associations between self‐esteem and depressive symptoms at each time point, as well as significant moderate stability paths. There were small reciprocal cross‐lagged associations, with associations from depressive symptoms to self‐esteem that were approximately one‐and‐a‐half times as strong as the other way around.

The cross‐lagged results of the within‐person RI‐CLPM and the CLPM showed that conclusions about the directionality of the association between self‐esteem and depressive symptoms would be somewhat different for the two models. The results of the within‐person analyses showed only a significant association from self‐esteem to depressive symptoms, and not the other way around, although the effect sizes were the same. The CLPM suggested significant reciprocal associations, with stronger associations from depressive symptoms to self‐esteem than the other way around. The model fit of the RI‐CLPM was better (i.e. higher CFI and lower RMSEA and SRMR) than that of the CLPM, indicating that teasing apart within‐person from between‐person effects in an RI‐CLPM matches more accurately with the actual data structure than assuming a blend of within‐person and between‐person variances in a more parsimonious CLPM approach.

## Study 2

### Methods

#### Sample and procedure

Participants for this study were recruited on T1 from grades 9 to 12 of three schools in Belgium (age range at T1: 13–20 years). We used the first three measurement waves out of a total of four, because the first three waves concerned the adolescent period and were therefore the most comparable with the other two studies. The measures took place one year apart between February 2009 and February 2011. Passive consent was obtained from parents, and adolescents could withdraw consent on the day of testing. Less than 1% of the possible sample did not obtain parental consent, and approximately 4% of the adolescents revoked consent. The study had a drop‐in design; participants who did not participate at T1 could enter the study at T2 or T3. A total of 1455 participants participated in the study on at least one of the measurement waves. From the participants at T2 and T3, respectively, 77% and 61% also participated on T1 (see Table 1 for descriptive information). Missing data were not completely at random as indicated by the significant Little's MCAR test (χ^2^ = 74.86, *df* = 42, *p* = .001). Subsequent exploration showed that boys were more likely to have missing data on all of the self‐esteem and depressive symptoms measures. In addition, lower levels of self‐esteem and higher levels of depressive symptoms were predictive for missing data at the next measurement wave. The FIML estimation was used to handle these missing data patterns. The questionnaires were administered in school classes during school hours, and packages with questionnaires were also sent to the homes of participants who graduated or left their school. Ethical approval for the study was obtained from the universities' institutional review board.

#### Measures

##### Self‐esteem

Self‐esteem was measured with 10 items of the Rosenberg Self‐esteem Scale (RSS; Rosenberg, 1965). Responses were indicated on a 4‐point scale ranging from 1 ‘*does not fit me at all*’ to 4 ‘*does fit me well*’. An example item is ‘On the whole, I am satisfied with myself’. Five negatively framed items were recoded in a way that higher scores indicate higher levels of self‐esteem on all items. The reliability of the RSS as indicated by Cronbach's alpha ranged from .88 to .89 across the three time points.

##### Depressive symptoms

Depressive symptoms were measured with the same 11 items of the Iowa CES‐D (T1–T3 *α* = .86–.87) as in study 1.

### Results

**Figure 4:**
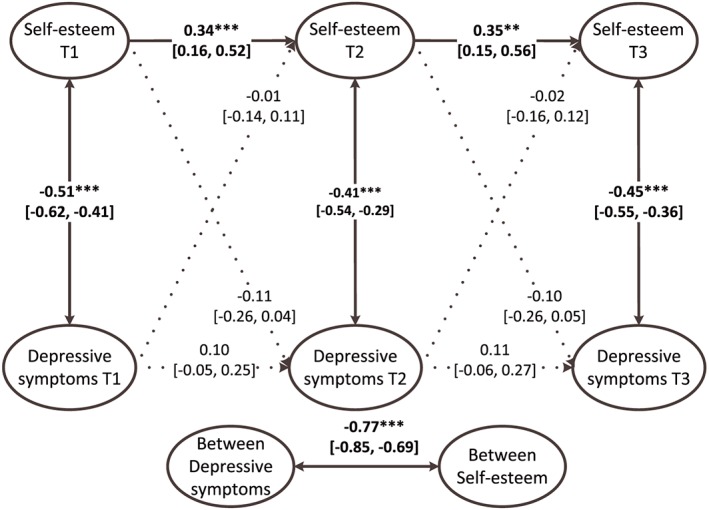
Simplified random intercept cross‐lagged panel model with standardized coefficients from study 2. Numbers between brackets indicate the 95% confidence interval. [Colour figure can be viewed at wileyonlinelibrary.com]

The ICCs indicated that 68% of the variance of self‐esteem could be explained by between‐person differences (32% by fluctuations within persons) and 53% of variance of depressive symptoms could be explained by between‐person differences (47% by fluctuations within persons). Model fit of the constrained RI‐CLPM was again excellent, χ^2^(5) = 2.10, *p* = .83, CFI = 1.000, RMSEA = 0.000, SRMR = 0.009 (unconstrained model fit χ^2^(1) = 1.19, *p* = .28, CFI = 1.00, RMSEA = 0.011, SRMR = 0.006). Standardized results are depicted in Figure 4 and unstandardized results in Table 2. There was a strong negative between‐person association between self‐esteem and depressive symptoms, indicating a strong trait‐like association. On the within‐person level, the results indicated significant within‐time correlations between self‐esteem and depressive symptoms. There were moderate significant within‐person carry‐over stability effects for self‐esteem but not for depressive symptoms. Thus, deviations from the expected self‐esteem score tended to carry over to the next measurement moment, while this was not the case for depressive symptoms. Importantly, for the cross‐lagged effects, the effect size of the effect from self‐esteem to depressive symptoms was larger than the other way around, although none of the cross‐lagged paths were significant.

**Figure 5:**
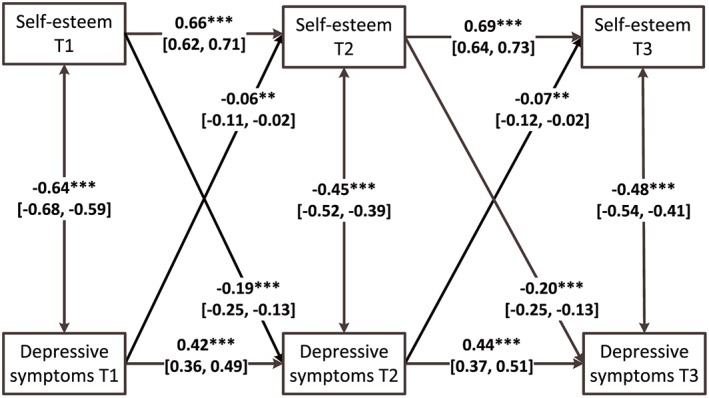
Cross‐lagged panel model with standardized coefficients from study 2. Numbers between brackets indicate the 95% confidence interval. [Colour figure can be viewed at wileyonlinelibrary.com]

The constrained CLPM had acceptable to good model fit, χ^2^(8) = 45.34, *p* < .001, CFI = 0.978, RMSEA = 0.057, SRMR = 0.042 (unconstrained model fit χ^2^(4) = 43.47, *p* < .001, CFI = 0.976, RMSEA = 0.082, SRMR = 0.039). Standardized results are depicted in Figure 5 and unstandardized results in Table 2. There were moderate to strong negative concurrent associations. There were significant negative small reciprocal cross‐lagged associations between depressive symptoms and self‐esteem, with around three times stronger associations from self‐esteem to depressive symptoms than the other way around.

The cross‐lagged results of the CLPM and RI‐CLPM differed notably. Whereas the CLPM, in which a blend of within‐person and between‐person variances is analysed, suggested significant reciprocal associations, weaker and nonsignificant cross‐lagged associations at the within‐person level were found with the RI‐CLPM. The model fit of the RI‐CLPM was again better (i.e. higher CFI and lower RMSEA and SRMR) than that of the CLPM, suggesting that the former model provides a better representation of the actual data structure than the latter.

## Study 3

### Methods

#### Sample and procedure

For this study, we used data collected at Rutgers University between 2008 and 2014. Data were collected at baseline, 18 months follow‐up, and 36 months follow‐up. For recruitment, information letters were sent to the homes of participating school districts around the central New Jersey area, of whom a child was in third, sixth, or ninth grade. Inclusion criteria for the study were that both parents and child were fluent in English, the child did not have an autism spectrum disorder or psychotic disorder, and the child had an IQ above 70. For a more detailed sample description, please see Hankin et al. (2015).

The final sample consisted of 316 children (age range at T1: 7–16 years). Caretakers had to provide written informed consent for the participation of the child, and the children written informed assent. Children visited the laboratory with their parents three times with 18 months intervals to fill in questionnaires and partake in other parts of the study. The retention rates were 86% for T2 and 77% for T3 (see Table 1 for descriptive information). Missing data were completely missing at random according to Little's MCAR test (χ^2^ = 15.43, *df* = 20, *p* = .75). Missing data were handled using FIML estimation. The research procedures were approved by the institutional review board of Rutgers University (08‐486Mc).

#### Measures

##### Self‐esteem

Self‐esteem was measured with five items of the RSS. The items were ‘on the whole I am satisfied with myself’, ‘at times I think I am no good at all’, ‘I feel that I have a number of good qualities’, ‘I am able to do things as well as most other people’, and ‘I feel I do not have much to be proud of’. Responses were indicated on a scale ranging from 1 ‘*strongly agree*’ to 4 ‘*strongly disagree*’, but scores were recoded so that higher scores indicated higher levels of self‐esteem. The reliability of this five‐item version of the RSS ranged from .72 to .84 across the three time points.

##### Depressive symptoms

Depressive symptoms during the previous two weeks were measured using 21 items of the Child Depression Inventory (CDI; Kovacs, 1985). The CDI is a widely used instrument to assess depressive symptoms in children and adolescents, and studies generally show good internal consistency and test–retest reliability (Klein, Dougherty, & Olino, 2005). To prevent overlap with the self‐esteem measure, six items of the original 27 CDI items that were independently judged to relate to self‐esteem by three raters were excluded. 2Items excluded: ‘I do most things ok’, ‘I hate myself’, ‘All bad things are my fault’, ‘I look ok’, ‘I can never be as good as the other kids’, and ‘Nobody really loves me’. Each item consisted of three statements indicating difference in severity of a symptom, scored from 0 to 2. An example item is ‘I do not feel alone’, ‘I feel alone many times’, and ‘I feel alone all the time’. The reliability of the 21‐item CDI ranged from .80 to .87 across the three time points.

### Results

**Figure 6:**
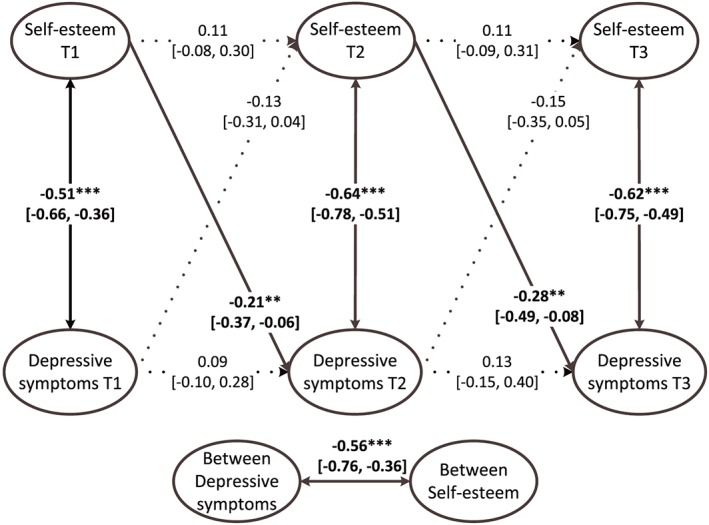
Simplified random intercept cross‐lagged panel model with standardized coefficients from study 3. Numbers between brackets indicate the 95% confidence interval. [Colour figure can be viewed at wileyonlinelibrary.com]

The ICCs for self‐esteem and depressive symptoms indicated that 47% of the variance of self‐esteem could be explained by between‐person differences (53% by fluctuations within persons) and 42% of variance of depressive symptoms could be explained by between‐person differences (58% by fluctuations within persons). The constrained RI‐CLPM again had an excellent model fit, χ^2^(5) = 2.93, *p* = .71, CFI = 1.000, RMSEA = 0.000, SRMR = 0.017 (unconstrained model fit χ^2^(1) = 2.60, *p* = .11, CFI = 0.997, RMSEA = 0.071, SRMR = 0.017). Standardized results are depicted in Figure 6 and unstandardized results in Table 2. There was a strong negative between‐person association between self‐esteem and depressive symptoms over time. At the within‐person level, significant negative concurrent associations were found between self‐esteem and depressive symptoms at each time point. There were small but not significant within‐person carry‐over stability effects for self‐esteem and depressive symptoms. There were significant cross‐lagged associations, at the within‐person level, from self‐esteem to depressive symptoms but not the other way around.

**Figure 7:**
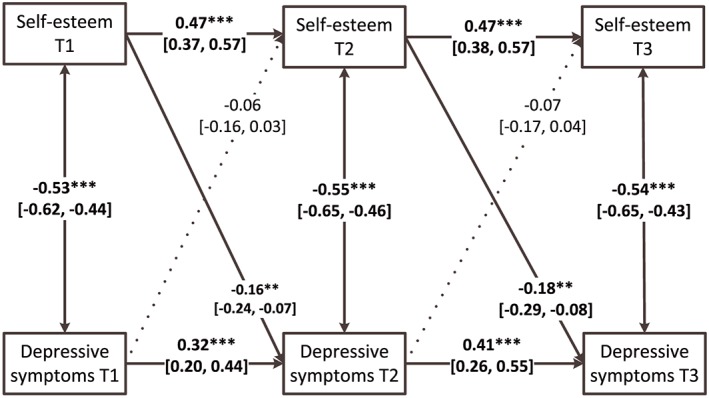
Cross‐lagged panel model with standardized coefficients from study 3. Numbers between brackets indicate the 95% confidence interval. [Colour figure can be viewed at wileyonlinelibrary.com]

The constrained CLPM, in which within‐person and between‐person variances are not teased apart, had acceptable model fit as indicated by the CFI and SRMR, but insufficient as indicated by the RMSEA, χ^2^(8) = 35.66, *p* < .001, CFI = 0.937, RMSEA = 0.105, SRMR = 0.051 (unconstrained model fit χ^2^(4) = 32.86, *p* < .001, CFI = 0.935, RMSEA = 0.151, SRMR = 0.047). Standardized results are depicted in Figure 7 and unstandardized results in Table 2. The results indicated strong cross‐sectional associations between self‐esteem and depressive symptoms at each time point. There were moderate stability effects for self‐esteem and depressive symptoms. There were small significant cross‐lagged associations from self‐esteem to depressive symptoms but no significant associations from depressive symptoms to self‐esteem.

The cross‐lagged results from the RI‐CLPM and CLPM were similar across the two models with regard to the pattern of results, with only significant and stronger effects from self‐esteem to depressive symptoms than the other way around in both models. However, all within‐person cross‐lagged effect sizes in the RI‐CLPM were larger than the cross‐paths in the CLPM (which represent a mixture of within‐person and between‐person variances). Moreover, the model fit of the RI‐CLPM was excellent, while it was not optimal for the CLPM, again highlighting that an RI‐CLPM provides a better description of the actual patterns in the data.

## Post Hoc Analyses

In our models, we examined associations over time between self‐esteem and depressive symptoms. A prerequisite to do so is that the used questionnaire items are answered and interpreted the same way over time. To examine whether self‐esteem and depressive symptoms scores could be meaningfully compared over the three measurement waves of the three studies, we tested for measurement invariance across these waves for the multi‐item self‐esteem and depressive symptoms measures. Following recommendations by Chen (2007), we considered metric invariance (i.e. invariance of factor loadings) as established when compared with the configural model, ∆CFI < 0.010, ∆RMSEA < 0.015, and ∆SRMR < 0.030, and scalar invariance (i.e. invariance of item intercepts) when compared with the metric model, ∆CFI < 0.010, ∆RMSEA < 0.015, and ∆SRMR < 0.010. Although not included in the recommendations of Chen (2007), we also tested for strict invariance (i.e. invariance of the item residuals), using the same change in model fit criteria as for scalar invariance. Measurement invariance could not be examined for the single‐item self‐esteem measure of study 1. We included correlated residuals within measurement waves if necessary and reasonable (e.g. between originally reverse coded items). The results of the analyses are presented in Table S2. Strict invariance, which implies that mean scores can be compared across time points, was established for the depressive symptom measures of studies 1 and 2 and the self‐esteem measure of study 2. Partial scalar invariance was established for the self‐esteem measure of study 3, with only one item that needed a freely estimated intercept at one time point. Although strictly spoken partial scalar invariance is not sufficient to compare mean scores, the bias will be limited. Measurement invariance of the depressive symptoms measure in study 3 could not be established, because the configural model had insufficient model fit. Although the fit was acceptable for RMSEA (0.045) and SRMR (0.075), it was below acceptable levels for the CFI (0.701). When looking at the null model, the RMSEA of the null model was 0.08, far below the recommended minimum of 0.158 for a null model (Kenny, 2015). A low RMSEA of the null model indicates that the CFI of the tested model is not informative. A possible reason for low RMSEA of the null model is that multiple items had no or very small correlations with some of the other items. In addition to measurement invariance over time, we also investigated measurement invariance within measurement waves in study 2. This study had large heterogeneity in age within measurement waves, with standard deviations larger than the time interval between measurement waves. On each measurement wave, the data were divided into a younger and older age group. We used models in which the same residuals were allowed to correlate as used in the measurement invariance analyses over time and added one extra pair of correlated residuals for the self‐esteem measure. The results showed strict invariance for the self‐esteem and depressive symptoms measures on all measurement moments (Table S3). Study 3 had large heterogeneity in age as well, but the small sample prevented us from conducting measurement invariance analyses within each measurement wave.

Our studies had substantial missing data due to attrition and the drop‐in designs in study 1 and study 2, which may have influenced the outcomes of the studies. We therefore conducted complete case analyses on the RI‐CLPM and CLPM models for all three studies to check the robustness of the findings. The results of the complete case analyses are included as supporting information (RI‐CLPM: Table S4; CLPM: Table S5). Although sample sizes dropped considerably, estimates and patterns were highly similar to the here reported results, speaking for the robustness of the findings.

Across the three studies, the model fit of the RI‐CLPM model was better than for the CLPM. To formally test the differences in model fit between the models, we performed Satorra–Bentler scaled chi‐squared tests and compared the Akaike information criterion (Akaike, 1974) and the Bayesian information criterion (Schwarz, 1978) of the models. The chi‐squared difference test was significant in all cases, and RI‐CLPM models always had lower Akaike information criterion and Bayesian information criterion values than the CLPM, indicating that RI‐CLPM models had a better model fit than the CLPM models (Table S6).

The results of the RI‐CLPMs and CLPMs across studies showed differences in significance level and effect size in all associations. Although these differences may reflect actual differences between the associations across studies or differences in power, the differences may also be due to random variation. Moreover, differences between significant and nonsignificant effects and differences in strength of the associations within the models, between models, and between studies are not necessarily significant in itself (Gelman & Stern, 2006). It is therefore difficult to infer general conclusions based on the individual studies. An advantage of multiple datasets is that the evidence of individual studies can be combined into meta‐analyses to get more robust findings. We therefore synthesized the results of the RI‐CLPMs and CLPMs over the three studies (total *N* = 3719) by conducting single‐paper meta‐analyses in spss 23 using the macro of Wilson (2005). Homogeneity analyses of the effect sizes across the three studies showed that for some of the estimates, it could not be assumed that the estimates of the individual studies reflect the same population effect size. This means that differences in effect sizes between studies were not only due to sampling error but also likely due to true differences in effect size. To account for the differences in population effect sizes between studies, we conducted separate random effect models for each standardized coefficient in the models. In these random effect models, studies were weighted by using the within‐study inversed sampling variance plus a constant representing the variability in variance across studies (Borenstein, Hedges, Higgins, & Rothstein, 2010). The small number of studies included in the meta‐analyses poses a limitation on the accuracy of the random effect estimates, which should therefore be considered as approximate estimates (Hedges & Vevea, 1998). 3Results of fixed effect models were highly similar. The output of the fixed effect models can be found alongside the random effect models on our OSF page: www.osf.io/p7xcj.


**Figure 8:**
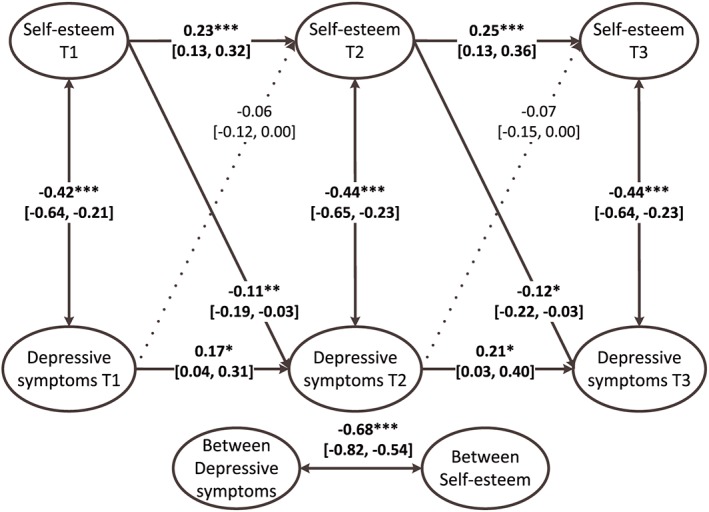
Standardized random intercept cross‐lagged panel model coefficients of the meta‐analysis over studies 1–3. Numbers between brackets indicate the 95% confidence interval. [Colour figure can be viewed at wileyonlinelibrary.com]

**Figure 9:**
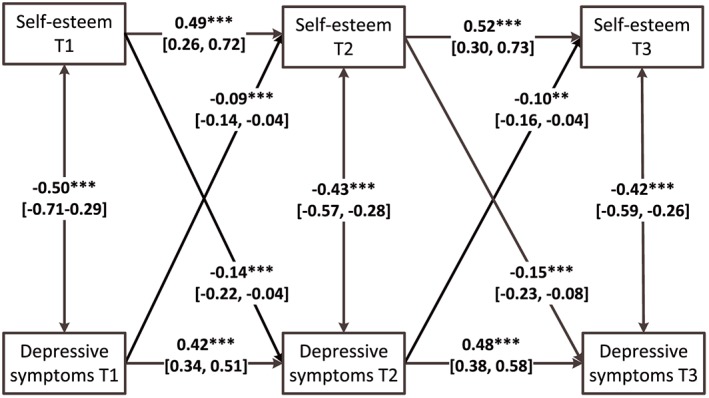
Standardized cross‐lagged panel model coefficients of the meta‐analysis over studies 1–3. Numbers between brackets indicate the 95% confidence interval. [Colour figure can be viewed at wileyonlinelibrary.com]

Aggregating the RI‐CLPM associations showed significant within‐person concurrent associations (Figure 8) and significant small within‐person carry‐over stability paths for both self‐esteem and depressive symptoms. Overall, participants who scored higher or lower than their expected self‐esteem and depressive symptoms scores tended to do so as well at the next time point. There were small (*β* = −.11 and *β* = −.12) significant and negative within‐person cross‐lagged effects from self‐esteem to depressive symptoms as well. Thus, participants' deviations from their expected depressive symptoms score could be predicted by deviations from their expected self‐esteem score at the previous time point, after controlling for deviations from the expected depressive symptoms score at the previous time point. The cross‐lagged effects from depressive symptoms to self‐esteem were smaller (*β* = −.06 and *β* = −.07) and not significant. The aggregated effects of the CLPM were all significant (Figure 9). The cross‐lagged associations from self‐esteem to depressive symptoms were somewhat stronger than the other way around (*β* = −.14 and *β* = −.15 vs *β* = −.09 and *β* = −.10). Thus, even though the RI‐CLPM isolates the within‐person effects from between‐person effects and the CLPM examines a mixture of both sources of variance, they showed similar cross‐lagged paths, with only small differences in effect sizes. However, the combined evidence across studies was not sufficient to reject the null hypothesis that there is no cross‐lagged association from depressive symptoms to self‐esteem in the RI‐CLPM, whereas this was the case in the CLPM. However, this difference is most likely due to substantial power differences between the two models. A detailed description of power calculations for the RI‐CLPM and CLPM is presented in Appendix A of the Supporting Information.

## Discussion

We investigated two hypotheses derived from the two most dominant theories describing the association between self‐esteem and depression. These are the vulnerability model (i.e. low self‐esteem makes one vulnerable to developing depression) and the scar model (i.e. going through depression is damaging for self‐esteem). We followed previous research by operationalizing a scar effect as a longitudinal negative effect of depressive symptoms on self‐esteem, without taking into consideration diagnostic status or whether depressive symptoms were still present on the last measurement moment. In order to replicate the results, we used three different datasets coming from three different countries, with measurement intervals of 1 year (studies 1 and 2) and 1.5 years (study 3). To provide more robust findings than the individual studies, results of the individual studies were aggregated using meta‐analysis. Importantly, we investigated the associations between self‐esteem and depressive symptoms on both the between‐person level and the within‐person level. We did so by using an RI‐CLPM (Hamaker et al., 2015) and compared the results with the most commonly used method so far, the regular CLPM (e.g. Orth, Robins, & Roberts, 2008; Orth, Robins, Trzesniewski, Maes, & Schmitt, 2009; Rieger et al., 2016). The main difference between the two approaches is that the former differentiates between‐person effects from within‐person effects, and the latter does not. By controlling for all stable confounders at the between‐person level and by analysing the level at which the actual processes are hypothesized to occur, the RI‐CLPM provides a much more stringent test of the within‐person processes described. Our study was inspired by research showing that results found in a CLPM do not always replicate at the level of within‐person, and can even be opposite in direction and strength, especially when the constructs under study are to some extent trait like (Dietvorst et al., 2017; Hamaker et al., 2015; Keijsers, 2016). Translating this to the current field of study, in which CLPM is a popular way of testing these hypotheses, we argue that evidence for the vulnerability model and the scar model needs to be re‐evaluated.

The between‐person results in the current study showed a strong trait effect for the negative association between self‐esteem and depressive symptoms in all three datasets. Individuals with high self‐esteem across the three measurement periods, compared with other individuals, also tended to have low levels of depressive symptoms over the measurement periods, compared with other individuals. This strong correlation could not be explained by content overlap in the measures, because items relating to self‐esteem were excluded from the depressive symptoms measures.

After controlling for these trait effects, consistent within‐person associations were found at each measurement wave and across all three studies, although the strength of the associations varied from small to strong (Cohen, 1992). These associations speak against the idea that self‐esteem and depressive symptoms form a common factor (Watson, Suls, & Haig, 2002), because the time‐invariant common factor has been excluded by the inclusion of random intercepts.

Before elaborating on the within‐person effects, it is useful to make a few remarks with regard to the issue of concept overlap between self‐esteem and depression. Although the two concepts are correlated and low self‐esteem is a symptom of depression, low self‐esteem does not only occur in the context of depression and neither does the presence of low self‐esteem suffice to diagnose depression. In other words, someone may have low self‐esteem but no other symptoms of depression, and a depressed person may not have low self‐esteem. Two independent studies showed that a one‐factor model had an inferior model fit than a model with separate factors for self‐esteem and depression (Orth, Robins, & Roberts, 2008; Rieger et al., 2016). Thus, although the concepts may correlate moderately to strongly, they are not indistinguishably related. Moreover, because content overlap was controlled for by excluding self‐esteem items from the depression measures and by controlling for any time‐invariant common factor via the random intercepts, we believe that the associations found represent relationships between non‐overlapping constructs.

The within‐person effects of most interest were the cross‐lagged effects in the RI‐CLPM, because these provide a critical test about how self‐esteem and depressive symptoms predict one another over time within persons, and about whether the results support the vulnerability model and the scar model. Overall, our within‐person effects showed a pattern that was similar to previous research (Sowislo & Orth, 2013), namely, significant vulnerability effects but no significant scar effects, and vulnerability effects which were almost twice as large as the scar effects. Of note, the aggregated results were not consistently found in the individual studies. Inconsistency in *p*‐values can be expected though, as even in case of a true effect, nonsignificant findings are to be expected when multiple studies are conducted (Lakens & Etz, 2017; Schimmack, 2012). The major advantage of the meta‐analyses was that we were able to synthesize the evidence from the individual studies, representing the combined evidential weight. We would like to stress that we do not interpret the nonsignificant scar effects in our study as evidence of absence of the scar effect, neither do we interpret the differences in effect size and significance level between the vulnerability and scar effects as proof that these effects are significantly different.

Together with the between‐person effects found in this study and previous research, the results provide valuable insight into the association between self‐esteem and depressive symptoms in adolescents. Adolescents with self‐esteem levels lower than their peers tend to be the ones who are also likely to experience more depressive symptoms. These between‐person results thus show who are most likely to need an intervention, compared with their peers. The within‐person results of our study further provide insight in how self‐esteem and depressive symptoms influence each other at the level of an individual adolescent. Adolescents who experience lower self‐esteem than they usually do are at risk of an increase in their depressive symptoms. The results also show that the effect sizes were small, suggesting that self‐esteem may not be a major risk factor for developing depressive symptoms over a 1‐ to 1.5‐year time period. The results should therefore not be over interpreted. Yet, if these small effects cascade over time, the influence of self‐esteem on depressive symptoms may be more substantial over multiple years; based on this study, we cannot conclude whether this is indeed the case. Further within‐person replications are needed before substantive statements about the nature of the within‐person association between self‐esteem and depressive symptoms can be made. If the results replicate in future studies, and if effects cascade over time, interventions aimed at self‐esteem enhancement in adolescents with low self‐esteem may be beneficial for reducing the risk for developing depressive symptoms. Based on experiences of self‐esteem enhancement programmes deployed in the past, it is probably most effective to focus on both self‐esteem itself and its antecedents such as formation of supportive relationships and academic competence (DuBois, Flay, & Fagen, 2009; O'Mara, Marsh, Craven, & Debus, 2006). Further replication studies could also extend the models by investigating moderating factors, as is, for example, proposed in the diathesis stress model. The diathesis stress model states that low self‐esteem is a vulnerability to developing depression only or especially under stressful conditions (Abela & Hankin, 2008).

Next to the within‐person cross‐lagged effects, which are relevant for testing the vulnerability model against the scar model, the RI‐CLPMs provided information about within‐person carry‐over stability effects (i.e. instances in which a person scores above or below his or her own expected scores are likely to be followed with a deviation on the next measurement). Aggregated findings across datasets showed significant small carry‐over stability effects for both self‐esteem and depressive symptoms. This suggests that, despite a relatively high rank‐order stability between persons (Robins & Trzesniewski, 2005), the carry‐over stability—within one person—is much smaller. The small carry‐over stability effects for self‐esteem and depressive symptoms are not surprising given the many social and physical challenges that adolescents face during this developmental period (Hankin, 2006), resulting in variability and fluctuation around an individual's usual self‐esteem and mood level (Maciejewski, van Lier, Branje, Meeus, & Koot, 2015). Whether these relatively small carry‐over effects are indeed characteristic for the fluctuations occurring during the developmental stage of adolescence has to be investigated by comparing the results with the outcomes of future studies using adult samples.

In addition to elucidating temporal associations between self‐esteem and depressive symptoms at the within‐person level, we also aimed to critically investigate whether our conclusions would have differed if the presumed within‐person associations were investigated with the commonly used CLPM (e.g. Orth et al., 2014; Orth, et al., 2016; Rieger et al., 2016), a method that does not separate between‐person effects from within‐person effects. After combining the three datasets, the conclusions based on the RI‐CLPM and CLPM with regard to the cross‐lagged paths were largely comparable: both approaches indicated the predominance of the vulnerability effect with quite comparable effect sizes. The CLPM indicated significant scar effects while the RI‐CLPM did not, but this difference in significance may reflect a difference in power rather than more substantial differences. Thus overall, with this specific research question, the within‐person processes seem to largely overlap with findings at the between‐person level. This is reassuring, in that the results of previous longitudinal studies may not be too far off from what would have been found if within‐person methods were used. However, there are several studies in which the within‐person process and the between‐person pattern of results are distinct, sometimes even opposing (Hamaker et al., 2015; Keijsers, 2016; Kievit, Frankenhuis, Waldorp, & Borsboom, 2013; Oerlemans, Rommelse, Buitelaar, & Hartman, 2018). Although this individual study indicates a convergence between the different levels of covariance, this means neither that results of previous research can now be safely interpreted in terms of within‐person effects nor that we can continue to rely on CLPM instead of within‐person methods like the RI‐CLPM when the goal is to examine within‐person processes (e.g. Berry & Willoughby, 2016). The theoretical reasons to use within‐person analyses, and the superior model fit of the RI‐CLPM compared with the CLPM, advocate the use of the RI‐CLPM. As a final note, we used the RI‐CLPM to separate within‐person effects from between‐person effects, but other models are available and may be better suited depending on the research question. A discussion of the different models is beyond the scope of this article, but there are several articles available for the interested reader (e.g. Bainter & Howard, 2016; Curran, Howard, Bainter, Lane, & McGinley, 2014; Hamaker et al., 2015).

### Limitations

The current study had several limitations. First, given that not all studies used the same measurement instruments, differences between studies may reflect differences in measurement instruments. In study 1, we had to rely on a single‐item measure of self‐esteem, which had a lower than desired estimated reliability that probably attenuated the stability coefficients. This single‐item measure is also less appropriate to treat as a continuous variable than the multi‐item self‐esteem measures as used in the other two studies. Moreover, measurement invariance of the CDI as used in study 3 could not be established because of insufficient model fit. Second, although it is a major strength of our study that we used three datasets, together covering the period from pre‐adolescence to early adulthood, the differences in age coverage and time intervals between the measurements also form a limitation. Although previous research suggests that vulnerability effects are stable across the lifespan (Orth et al., 2009), it is possible that within‐person dynamics do change over time during the transitionary period of adolescence. If this is the case, aggregating data across studies may distort knowledge about the within‐person associations instead of strengthening it. In addition, study 3 had 1.5‐year time intervals between the measurement waves, as opposed to the 1‐year time intervals in the other two studies, which hampers comparability of effect sizes across the three studies. Although it is rather common to have studies included in meta‐analyses that do not have the same time interval between measures, after accounting for trait effects, effects likely manifest themselves differently across different time intervals, making it possibly inappropriate to combine the effects (Dormann & Griffin, 2015; Voelkle, Oud, Davidov, & Schmidt, 2012). Moreover, most within‐person interactions between self‐esteem and depressive symptoms are likely to occur on a much smaller timescale than the 1‐ to 1.5‐year intervals used in the three studies presented here. That is, self‐esteem and depressive symptoms are likely to influence each other on a timescale of weeks, days, or even smaller, instead of years (Dormann & Griffin, 2015). This may partly explain the rather small cross‐lagged effect sizes and the comparatively strong concurrent effects. Possibly, over‐time effects that occurred on a shorter time interval were not captured by the cross‐lagged paths but instead ended up in the concurrent associations. Studies investigating within‐person associations between self‐esteem and depressive symptoms on a much smaller timescale are needed. A particularly useful approach is to measure associations in daily life using experience sampling methods. More extensive data collection has the additional advantage that more measures can provide more reliable within‐person estimates than the three measurement waves we currently used. Another question to further investigate is whether the optimal time intervals between measurement waves differ for vulnerability and scar effects, with longer expected optimal time intervals for scar effects. Third, all three studies had substantial missing data due to attrition and the drop‐in designs used in studies 1 and 2, in which participants were allowed to enter the study after the first measurement wave. In studies 1 and 2, boys were more likely to have missing data and in study 2, participants with low self‐esteem or high levels of depressive symptoms as well. However, the FIML estimation we used is quite capable of handling missing data under missing at random assumptions, thus limiting the potential distorting of the estimates. The robustness of the findings was supported by the post hoc complete case analyses, which yielded highly similar results. Nevertheless, power analyses showed that, under the assumption that the meta‐analysed effects give an approximation of the true effect size, none of the individual studies were powered sufficiently to detect the vulnerability or scar effects. This highlights the need for large samples and the use of meta‐analytical techniques to combine power and evidence across studies, as we presented in the current study. Fourth, despite the many advantages of the RI‐CLPM over a CLPM, it has some limitations as well. The RI‐CLPM does not take measurement error into account. Measurement error in the variables included has probably attenuated the effects in some unknown degree. However, extending the model to account for measurement error tends to make the model difficult to estimate and may require more than 10 measurement waves (Hamaker et al., 2015). Another point to keep in mind is that the reported within‐person effects in the RI‐CLPMs reflect averaged within‐person effects. There may be considerable within‐person heterogeneity in the processes that link self‐esteem to depression, and the average effect may not be representative for many individuals. Investigation of models with random effects for the structural parts of the model or person‐specific analyses is needed to determine whether it is warranted to generalize across individuals. In addition, the RI‐CLPM can be used to separate between‐person effects from within‐person effects and to examine the within‐person effects prospectively. However, on the between‐person level, only between‐person associations over all time points are estimated; it is thus not possible to examine prospective between‐person effects with the RI‐CLPM. Fifth, we were only able to test the commonly used simplified operationalization of a scar model, instead of testing a true scar model (Ormel et al., 2004), that is, to test whether going through a depression has long‐lasting negative effects on self‐esteem even after the depression has remitted. Our findings thus do not preclude the possibility that going through a full‐blown depression does have long‐lasting negative effects on self‐esteem. Finally, our studies were conducted in three Western countries and in mostly adolescent samples; therefore, it is unclear whether the results would generalize to other cultures and age groups.

## Conclusion

Our study contributes to existing research examining longitudinal associations between self‐esteem and depressive symptoms in adolescents, by examining these associations within persons. The within‐person analyses were in line with the within‐person hypotheses that underlie the vulnerability model and scar model and were performed by means of an RI‐CLPM. Inspired by increasing critiques on the common use of CLPM for studying within‐person questions, we also examined whether results of CLPMs would differ from the within‐person analyses. Although the RI‐CLPM results largely converged with the CLPM results and previously found CLPM results (Sowislo & Orth, 2013), we advise against using the CLPM on theoretical and practical grounds. When within‐person effects were separated from between‐person effects, we found evidence for small within‐person effects. These small within‐person results were in line with the vulnerability model: adolescents were thus somewhat more vulnerable for developing depressive symptoms in and following periods with low self‐esteem. Although we found no support for the scar model, we did not test differences between the vulnerability and scar effects. The results can therefore not be interpreted as providing significantly more support for the vulnerability model than the scar model. At the between‐person level, adolescents with low self‐esteem over time were also the ones with more depressive symptoms over time (compared with other adolescents). The results are in need of further replication and extension. Important topics for future studies would be to investigate whether the dynamics of the association between self‐esteem and depressive symptoms are stable from early adolescence to early adulthood, to investigate the associations in daily life, to investigate individual differences in the lagged effects and whether these differences are related to person characteristics, and to investigate whether the results replicate in non‐Western cultures. Overall, our results show that low self‐esteem has a significant but rather small negative influence on depressive symptoms over time during adolescence.

## Supporting information

Table S1. Variance–Covariance Matrices Study 1–3
Table S2. Measurement Invariance Models Study 1–3
Table S3. Measurement Invariance Within Measurement Waves Study 2
Table S4. Standardized RI‐CLPM Estimates Complete Case Analyses Study 1–3
Table S5. Standardized CLPM estimates Complete Case Analyses Study 1–3
Table S6. Model Fit Comparisons of RI‐CLPM and CLPM Across Study 1–3
Table S6. Power Analyses RI‐CLPM and CLPM With and Without Missing Data

Open Practices Disclosure
