# Supplementary material for: The Longitudinal Association Between Self‐esteem and Depressive Symptoms in Adolescents: Separating Between‐Person Effects from Within‐Person Effects
Source: Eur J Pers. 2018 Nov 5;32(6):653–71. doi: 10.1002/per.2179 (PMC6519152; doi:10.1002/per.2179)
Supplement: Supplementary file 1 — Table S1. Variance–Covariance Matrices Study 1–3 Table S2. Measurement Invariance Models Study 1–3 Table S3. Measurement Invariance Within Measurement Waves Study 2 Table S4. Standardized RI‐CLPM Estimates Complete Case Analyses Study 1–3 Table S5. Standardized CLPM estimates Complete Case Analyses Study 1–3 Table S6. Model Fit Comparisons of RI‐CLPM and CLPM Across Study 1–3 Table S6. Power Analyses RI‐CLPM and CLPM With and Without Missing Data [file PER-32-653-s001.docx]

**Supplemental material**

Table S1. *Variance-Covariance Matrices Study 1-3*

|  | 1 | 2 | 3 | 4 | 5 | 6 |
| --- | --- | --- | --- | --- | --- | --- |
| **Study 1** |  |  |  |  |  |  |
| Self-esteem T1 (1) | 1.21 |  |  |  |  |  |
| Self-esteem T2 (2) | 0.49 | 1.45 |  |  |  |  |
| Self-esteem T3 (3) | 0.35 | 0.62 | 1.37 |  |  |  |
| Depressive symptoms T1 (4) | -0.15 | -0.13 | -0.12 | 0.18 |  |  |
| Depressive symptoms T2 (5) | -0.11 | -0.21 | -0.16 | 0.11 | 0.22 |  |
| Depressive symptoms T3 (6) | -0.09 | -0.17 | -0.21 | 0.09 | 0.13 | 0.21 |
| **Study 2** |  |  |  |  |  |  |
| Self-esteem T1 (1) | 0.35 |  |  |  |  |  |
| Self-esteem T2 (2) | 0.24 | 0.34 |  |  |  |  |
| Self-esteem T3 (3) | 0.21 | 0.24 | 0.32 |  |  |  |
| Depressive symptoms T1 (4) | -0.20 | -0.15 | -0.13 | 0.27 |  |  |
| Depressive symptoms T2 (5) | -0.14 | -0.18 | -0.14 | 0.15 | 0.28 |  |
| Depressive symptoms T3 (6) | -0.12 | -0.15 | -0.19 | 0.13 | 0.15 | 0.28 |
| **Study 3** |  |  |  |  |  |  |
| Self-esteem T1 (1) | 0.27 |  |  |  |  |  |
| Self-esteem T2 (2) | 0.14 | 0.28 |  |  |  |  |
| Self-esteem T3 (3) | 0.12 | 0.15 | 0.29 |  |  |  |
| Depressive symptoms T1 (4) | -0.06 | -0.04 | -0.04 | 0.05 |  |  |
| Depressive symptoms T2 (5) | -0.05 | -0.09 | -0.05 | 0.03 | 0.07 |  |
| Depressive symptoms T3 (6) | -0.02 | -0.05 | -0.07 | 0.02 | 0.03 | 0.05 |

Table S2. *Measurement Invariance Models Study 1-3*

|  | Model | χ^2^ | DF | Δ χ^2^ | RMSEA | Δ RMSEA | CFI | Δ CFI | SRMR | Δ SRMR |
| --- | --- | --- | --- | --- | --- | --- | --- | --- | --- | --- |
| Study 1 |  |  |  |  |  |  |  |  |  |  |
| Depressive symptoms | configural^a^ | 2099.36 | 481 |  | 0.042 |  | 0.862 |  | 0.052 |  |
|  | configural^b^ | 1061.35 | 478 |  | 0.025 |  | 0.950 |  | 0.046 |  |
|  | metric | 1127.59 | 500 | 66.24 | 0.025 | 0 | 0.946 | -0.004 | 0.051 | 0.005 |
|  | scalar | 1206.14 | 522 | 78.55 | 0.026 | 0.001 | 0.941 | -0.005 | 0.051 | 0 |
|  | strict | 1264.84 | 544 | 58.70 | 0.026 | 0 | 0.938 | -0.003 | 0.053 | 0.002 |
| Study 2 |  |  |  |  |  |  |  |  |  |  |
| self-esteem | configural^a^ | 1589.28 | 392 |  | 0.046 |  | 0.902 |  | 0.051 |  |
|  | configural^b^ | 1203.9 | 389 |  | 0.038 |  | 0.933 |  | 0.049 |  |
|  | metric | 1224.8 | 409 | 20.90 | 0.037 | -0.001 | 0.933 | 0 | 0.052 | 0.003 |
|  | scalar | 1291.15 | 429 | 66.35 | 0.037 | 0 | 0.930 | -0.003 | 0.056 | 0.004 |
|  | strict | 1337.51 | 449 | 46.36 | 0.037 | 0 | 0.927 | -0.003 | 0.064 | 0.008 |
| Depressive symptoms | |  |  |  |  |  |  |  |  |  |
|  | configural^a^ | 1880.47 | 481 |  | 0.045 |  | 0.866 |  | 0.054 |  |
|  | configural^b^ | 1003.52 | 478 |  | 0.028 |  | 0.950 |  | 0.050 |  |
|  | metric | 1045.92 | 500 | 42.40 | 0.027 | -0.001 | 0.948 | -0.002 | 0.053 | 0.003 |
|  | scalar | 1108.51 | 522 | 62.59 | 0.028 | 0.001 | 0.944 | -0.004 | 0.053 | 0 |
|  | strict | 1157.84 | 544 | 49.33 | 0.028 | 0 | 0.941 | -0.003 | 0.055 | 0.002 |
| Study 3 |  |  |  |  |  |  |  |  |  |  |
| self-esteem | configural^a^ | 134.23 | 82 |  | 0.045 |  | 0.949 |  | 0.041 |  |
|  | metric | 143.78 | 92 | 9.55 | 0.042 | -0.003 | 0.949 | 0 | 0.067 | 0.026 |
|  | scalar | 173.91 | 102 | 30.13 | 0.047 | 0.005 | 0.929 | -0.02 | 0.063 | -0.004 |
|  | partial scalar | 156.69 | 101 | 12.91 | 0.042 | 0 | 0.945 | -0.004 | 0.058 | -0.009 |
|  | strict | 213.89 | 111 | 57.20 | 0.054 | 0.012 | 0.899 | -0.046 | 0.118 | 0.060 |
| ^a^ Configural model where there are no within time point correlated residuals  ^b^ Configural model where within time point correlated residuals are allowed. Measurement invariance comparisons were made with this model as baseline when this model was included. See www.osf.io/p7xcj for a full list of the correlated risuduals. | | | | | | | | | | |

Table S3. *Measurement Invariance Within Measurement Waves Study 2*

|  | Wave (age group sizes) | Model | χ^2^ | DF | Δ χ^2^ | RMSEA | Δ RMSEA | CFI | Δ CFI | SRMR | Δ SRMR |
| --- | --- | --- | --- | --- | --- | --- | --- | --- | --- | --- | --- |
| Depressive symptoms | T1 (399/536) | configural^a^ | 166.37 | 82 |  | 0.047 |  | 0.967 |  | 0.034 |  |
|  |  | metric | 181.26 | 96 | 14.89 | 0.044 | -0.003 | 0.966 | -0.001 | 0.05 | 0.016 |
|  |  | scalar | 197.07 | 106 | 15.81 | 0.043 | -0.001 | 0.964 | -0.002 | 0.051 | 0.001 |
|  |  | strict | 209.19 | 117 | 12.12 | 0.041 | -0.002 | 0.964 | 0 | 0.055 | 0.004 |
|  | T2 (498/558) | configural^a^ | 211.24 | 82 |  | 0.055 |  | 0.958 |  | 0.040 |  |
|  |  | metric | 239.40 | 96 | 28.16 | 0.053 | -0.002 | 0.953 | -0.005 | 0.051 | 0.011 |
|  |  | scalar | 261.26 | 106 | 21.86 | 0.053 | 0 | 0.949 | -0.004 | 0.052 | 0.001 |
|  |  | strict | 268.55 | 117 | 7.29 | 0.050 | -0.003 | 0.950 | 0.001 | 0.055 | 0.003 |
|  | T3 (536/396) | configural^a^ | 146.90 | 82 |  | 0.041 |  | 0.977 |  | 0.033 |  |
|  |  | metric | 161.94 | 96 | 15.04 | 0.038 | -0.003 | 0.977 | 0 | 0.046 | 0.013 |
|  |  | scalar | 184.84 | 106 | 22.9 | 0.040 | 0.002 | 0.972 | -0.005 | 0.049 | 0.003 |
|  |  | strict | 209.67 | 117 | 24.83 | 0.041 | 0.001 | 0.967 | -0.005 | 0.056 | 0.007 |
| Self-esteem | T1 (398/539) | configural^a^ | 244.40 | 62 |  | 0.079 |  | 0.944 |  | 0.043 |  |
|  |  | metric | 255.46 | 76 | 11.06 | 0.071 | -0.008 | 0.944 | 0 | 0.053 | 0.010 |
|  |  | scalar | 273.54 | 86 | 18.08 | 0.068 | -0.003 | 0.942 | -0.002 | 0.056 | 0.003 |
|  |  | strict | 281.28 | 96 | 7.74 | 0.064 | -0.004 | 0.943 | 0.001 | 0.064 | 0.008 |
|  | T2 (502/560) | configural^a^ | 291.45 | 62 |  | 0.083 |  | 0.937 |  | 0.048 |  |
|  |  | metric | 303.66 | 76 | 12.21 | 0.075 | -0.008 | 0.937 | 0 | 0.055 | 0.007 |
|  |  | scalar | 316.58 | 86 | 12.92 | 0.071 | -0.004 | 0.937 | 0 | 0.057 | 0.002 |
|  |  | strict | 320.99 | 96 | 4.41 | 0.066 | -0.005 | 0.938 | 0.001 | 0.058 | 0.001 |
|  | T3 (539/394) | configural^a^ | 224.49 | 62 |  | 0.075 |  | 0.944 |  | 0.043 |  |
|  |  | metric | 236.96 | 76 | 12.47 | 0.067 | -0.008 | 0.945 | 0.001 | 0.055 | 0.012 |
|  |  | scalar | 253.55 | 86 | 16.59 | 0.065 | -0.002 | 0.943 | -0.002 | 0.061 | 0.006 |
|  |  | strict | 258.96 | 96 | 5.41 | 0.060 | -0.005 | 0.944 | 0.001 | 0.071 | 0.010 |
| ^a^ Configural model where correlated residuals were allowed. See osf.io/cxc6f for a full list of the correlated risuduals. | | | | | | | | | | | |

Table S4. *Standardized RI-CLPM Estimates Complete Case Analyses Study 1-3*

|  | **Study 1 (N = 748)** | | **Study 2 (N = 523)** | | **Study 3 (N = 238)** | | |
| --- | --- | --- | --- | --- | --- | --- | --- |
| **Estimated path** | **β [95% CI] (SE)** | **p-value** | **β [95% CI] (SE)** | **p-value** | **β [95% CI] (SE)** | **p-value** | |
| S T1 → S T2 | 0.23 [0.12, 0.34] (0.06) | < .001 | 0.34 [0.16, 0.52] (0.09) | < .001 | 0.09 [-0.10, 0.28] (0.10) | .352 | |
| S T2 → S T3 | 0.26 [0.14, 0.38] (0.06) | < .001 | 0.36 [0.15, 0.56] (0.10) | .001 | 0.09 [-0.11, 0.30] (0.10) | .371 | |
| D T1 → D T2 | 0.25 [0.14, 0.35] (0.05) | < .001 | 0.09 [-0.07, 0.25] (0.08) | .261 | 0.07 [-0.12, 0.26] (0.10) | .483 | |
| D T2 → D T3 | 0.31 [0.17, 0.44] (0.07) | < .001 | 0.09 [-0.08, 0.26] (0.09) | .288 | 0.10 [-0.18, 0.38] (0.14) | .481 | |
| S T1 → D T2 | -0.11 [-0.19, -0.04] (0.04) | .004 | -0.10 [-0.25, 0.06] (0.08) | .232 | -0.22 [-0.37, -0.07] (0.08) | .004 | |
| S T2 → D T3 | -0.13 [-0.22, -0.04] (0.05) | .004 | -0.09 [-0.25, 0.06] (0.08) | .246 | -0.29 [-0.50, -0.09] (0.11) | .005 | |
| D T1 → S T2 | -0.06 [-0.15, 0.03] (0.05) | .163 | -0.01 [-0.14, 0.11] (0.06) | .837 | -0.13 [-0.31, 0.04] (0.09) | .141 | |
| D T2 → S T3 | -0.08 [-0.19, 0.03] (0.06) | .157 | -0.01 [-0.15, 0.12] (0.07) | .838 | -0.15 [-0.36, 0.06] (0.11) | .159 | |
| S T1 ↔ D T1 | -0.18 [-0.30, -0.05] (0.07) | < .001 | -0.51 [-0.64, 0.39] (0.07) | < .001 | -0.49 [-0.65, -0.34] (0.08) | < .001 | |
| S T2 ↔ D T2 | -0.36 [-0.45, -0.26] (0.05) | .007 | -0.44 [-0.57, -0.30] (0.07) | < .001 | -0.66 [-0.80, -0.52] (0.07) | < .001 | |
| S T3 ↔ DT3 | -0.26 [-0.36, -0.16] (0.05) | < .001 | -0.47 [-0.58, -0.36] (0.06) | < .001 | -0.61 [-0.75, -0.48] (0.07) | < .001 | |
| Between person S ↔ D | -0.62 [-0.68, -0.36] (0.12) | < .001 | -0.75 [-0.88, -0.66] (0.05) | < .001 | -0.59 [-0.77, -0.41] (0.09) | < .001 | |
| Note. S = self-esteem; D = depressive symptoms | | | | | | |  |

Table S5. *Standardized CLPM estimates Complete Case Analyses Study 1-3*

|  | **Study 1 (N = 748)** | | **Study 2 (N = 523)** | | **Study 3 (N = 238)** | |
| --- | --- | --- | --- | --- | --- | --- |
| **Estimated path** | ***β* [95% CI] (SE)** | **p value** | ***β* [95% CI] (SE)** | **p value** | ***β* [95% CI] (SE)** | **p value** |
| S T1 → S T2 | 0.35 [0.29, 0.41] (0.03) | < .001 | 0.67 [0.64, 0.72] (0.03) | < .001 | 0.47 [0.36, 0.57] (0.05) | < .001 |
| S T2 → S T3 | 0.38 [0.31, 0.44] (0.03) | < .001 | 0.69 [0.63, 0.74] (0.03) | < .001 | 0.48 [0.37, 0.58] (0.05) | < .001 |
| D T1 → D T2 | 0.45 [0.39, 0.51] (0.03) | < .001 | 0.41 [0.33, 0.49] (0.04) | < .001 | 0.31 [0.19, 0.43] (0.06) | < .001 |
| D T2 → D T3 | 0.51 [0.44, 0.57] (0.03) | < .001 | 0.41 [0.33, 0.50] (0.05) | < .001 | 0.39 [0.24, 0.54] (0.08) | < .001 |
| S T1 → D T2 | -0.11 [-0.16, -0.07] (0.02) | < .001 | -0.18 [-0.25, -0.11] (0.04) | < .001 | -0.17 [-0.26, -0.08] (0.05) | < .001 |
| S T2 → D T3 | -0.12 [-0.18, -0.07] (0.03) | < .001 | -0.18 [-0.25, -0.11] (0.04) | < .001 | -0.20 [-0.31, -0.09] (0.05) | < .001 |
| D T1 → S T2 | -0.12 [-0.17, 0.07] (0.03) | < .001 | -0.06 [-0.11, -0.00] (0.03) | .038 | -0.06 [-0.16, 0.03] (0.05) | .205 |
| D T2 → S T3 | -0.14 [-0.19, 0.08] (0.03) | < .001 | -0.06 [-0.12, -0.00] (0.03) | .039 | -0.07 [-0.17, 0.04] (0.05) | .210 |
| S T1 ↔ D T1 | -0.30 [-0.37, -0.23] (0.04) | < .001 | -0.63 [-0.70, -0.57] (0.03) | < .001 | -0.54 [-0.64, -0.43] (0.05) | < .001 |
| S T2 ↔ D T2 | -0.35 [-0.42, -0.28] (0.04) | < .001 | -0.47 [-0.55, -0.39] (0.04) | < .001 | -0.56 [-0.66, -0.46] (0.05) | < .001 |
| S T3 ↔ DT3 | -0.28 [-0.36, -0.19] (0.04) | < .001 | -0.49 [-0.58, -0.40] (0.04) | < .001 | -0.53 [-0.65, -0.42] (0.06) | < .001 |
| Note. S = self-esteem; D = depressive symptoms | | | | | | |

Table S6. *Model Fit Comparisons of RI-CLPM and CLPM Across Study 1-3*

|  |  |  |  |  |  |  |  |  |
| --- | --- | --- | --- | --- | --- | --- | --- | --- |
|  | **Scaling Correction factor RI-CLPM / CLPM** | **df**  **RI-CLPM / CLPM** | **χ^2^**  **RI-CLPM / CLPM** | **Satorra-Bentler Scaled Chi Square Difference** | **df difference** | **p-value** | **AIC RI-CLPM/CLPM** | **BIC RI-CLPM/CLPM** |
| Study 1 | 1.276 / 1.271 | 5 / 8 | 4.63 / 30.18 | 25.71 | 3 | < .001 | 15827 / 15853 | 15949 / 15959 |
| Study 2 | 1.121 / 1.196 | 5 / 8 | 2.10 / 45.34 | 39.27 | 3 | < .001 | 6984 / 7030 | 7100 / 7130 |
| Study 3 | 0.953 / 1.010 | 5 / 8 | 2.93 / 35.66 | 30.08 | 3 | < .001 | 667 / 695 | 750 / 766 |

**Appendix A. Power analyses**

For the novel RI-CLPM, there have not yet been any published power analyses. The goal of the power analyses we present here is to provide a first indication of the power of the RI-CLPM compared to the CLPM. For the analyses we conducted Monte Carlo simulations for the RI-CLPM and CLPM using Mplus 8.0. To estimate the models, effect sizes for all the paths in the models had to be specified. Since we are the first to investigate the associations between self-esteem and depressive symptoms within persons using the RI-CLPM, our study provides the best source of information with regard to the within-person estimates. We therefore used the estimates provided by our meta-analyzed RI-CLPM results, disregarding significance level, as input for generating realistic datasets for the power analysis of both the RI-CLPM and the CLPM, to compare the power of the RI-CLPM and CLPM as best as possible. The analyses are provided with and without missing data patterns, because missing data influences power. For the missing data pattern, we used the weighted average missing data percentage at each time-point across the three studies (i.e. T1 33%, T2 28% and T3 29%). We note that in our power analyses, the pattern of missingness introduced was completely at random, this means that, (1) for example, self-esteem at T1 could be missing for a case, while depressive symptoms at T1 was not, and (2) having missing data at one wave also had no influence on the probability of having a missing value on the other measurement waves.

**Results, Conclusions and Recommendations**

The results of the power analyses are presented in Table S6. Both the CLPM and the RI-CLPM are well-able to detect small sized carry over stability effect (effect .21 or stronger), with sample sizes of 1000, even with missing data. The results also indicate that for small cross-lagged effects (i.e., standardized effects between .20-.50; Cohen, 1992), larger sample sizes are needed to have sufficient power in the RI-CLPM. In fact, in our study, assuming that the vulnerability effects of -.11 and -.12 are reliable estimates of the true population effect, 1500 to 2000 cases are needed to have sufficient power (i.e., >.80) to detect these effects with a three wave RI-CLPM model. Analyzing the same data with a CLPM, 1000 cases would be enough to detect these specified small vulnerability effects. What can also be observed is that effects of .07 or smaller (as the scar effect in our findings), need over 4000 cases in a RI-CLPM and 2000-2500 cases in a CLPM. When a missing data pattern is introduced, as is likely in real life data, power drops severely. Over 4000 cases would be needed to find small vulnerability effects with a RI-CLPM and 1500-2000 cases with a CLPM. Scar effects would be unlikely to be found with a RI-CLPM with even extremely large samples and a CLPM would also require a sample of 4000.

In general these findings hint that both CLPM and RICLPM are large sample techniques. In general, the RI-CLPM has lower power than a CLPM, but it is important to weigh this limitation against the strength of obtaining more valid estimates (see Hamaker et al., 2015) at the within-person level. These estimates do not necessarily generalize to other studies, as power is dependent on the size of the effects, the distribution of variance across ecological levels, the number of measurement waves - the more the better - and the amount of missing data. In fact, each study would require a Monte Carlo simulation to obtain power estimates before the design of the study. We share the syntaxes for conducting the Monte Carlo studies with RI-CLPM on the OSF page to facilitate this (www.osf.io/p7xcj).

Moreover, in the current study, we provide a means to meta-analyze the results of RI-CLPMs of different studies to obtain sufficient power for drawing accurate conclusions.

Table S6. Power Analyses RI-CLPM and CLPM With and Without Missing Data

|  | **Without missing data** | | | | | | | |
| --- | --- | --- | --- | --- | --- | --- | --- | --- |
|  | **T1-T2** | **T2-T3** | **T1-T2** | **T2-T3** | **T1-T2** | **T2-T3** | **T1-T2** | **T2-T3** |
|  | **self-self (.23)** | **self-self (.25)** | **depr-depr (.17)** | **depr-depr (.21)** | **self-depr (-.11)** | **self-depr (-.12)** | **depr-self (-.06)** | **depr-self (-.07)** |
| RI-CLPM 1000 | 0.998 | 0.999 | 0.958 | 0.993 | 0.493 | 0.615 | 0.190 | 0.254 |
| 1500 | 1 | 1 | 0.995 | 1 | 0.653 | 0.784 | 0.260 | 0.354 |
| 2000 | 1 | 1 | 0.999 | 1 | 0.777 | 0.890 | 0.334 | 0.462 |
| 2500 | 1 | 1 | 1 | 1 | 0.860 | 0.946 | 0.396 | 0.548 |
| 3000 | 1 | 1 | 1 | 1 | 0.915 | 0.973 | 0.467 | 0.624 |
| 3500 | 1 | 1 | 1 | 1 | 0.950 | 0.987 | 0.527 | 0.687 |
| 4000 | 1 | 1 | 1 | 1 | 0.973 | 0.995 | 0.577 | 0.746 |
| CLPM 1000 | 1 | 1 | 0.998 | 1 | 0.885 | 0.931 | 0.410 | 0.524 |
| 1500 | 1 | 1 | 1 | 1 | 0.971 | 0.989 | 0.567 | 0.698 |
| 2000 | 1 | 1 | 1 | 1 | 0.993 | 0.999 | 0.683 | 0.813 |
| 2500 | 1 | 1 | 1 | 1 | 0.999 | 0.999 | 0.782 | 0.891 |
| 3000 | 1 | 1 | 1 | 1 | 1 | 1 | 0.850 | 0.940 |
| 3500 | 1 | 1 | 1 | 1 | 1 | 1 | 0.901 | 0.968 |
| 4000 | 1 | 1 | 1 | 1 | 1 | 1 | 0.981 | 1 |
|  | **With missing data (T1 33%, T2 28% and T3 29%)** | | | | | | | |
|  | **T1-T2** | **T2-T3** | **T1-T2** | **T2-T3** | **T1-T2** | **T2-T3** | **T1-T2** | **T2-T3** |
|  | **self-self (.23)** | **self-self (.25)** | **depr-depr (.17)** | **depr-depr (.21)** | **self-depr (-.11)** | **self-depr (-.12)** | **depr-self (-.06)** | **depr-self (-.07)** |
| RI-CLPM 1000 | 0.914 | 0.950 | 0.703 | 0.857 | 0.245 | 0.314 | 0.109 | 0.130 |
| 1500 | 0.981 | 0.992 | 0.857 | 0.957 | 0.336 | 0.437 | 0.147 | 0.178 |
| 2000 | 0.996 | 0.998 | 0.941 | 0.989 | 0.423 | 0.550 | 0.172 | 0.225 |
| 2500 | 1 | 1 | 0.974 | 0.997 | 0.509 | 0.649 | 0.199 | 0.278 |
| 3000 | 1 | 1 | 0.988 | 1 | 0.580 | 0.731 | 0.234 | 0.324 |
| 3500 | 1 | 1 | 0.996 | 1 | 0.651 | 0.791 | 0.263 | 0.368 |
| 4000 | 1 | 1 | 0.998 | 1 | 0.713 | 0.841 | 0.297 | 0.407 |
| CLPM 1000 | 0.994 | 0.999 | 0.913 | 0.988 | 0.587 | 0.695 | 0.227 | 0.300 |
| 1500 | 1 | 1 | 0.985 | 1 | 0.758 | 0.852 | 0.307 | 0.416 |
| 2000 | 1 | 1 | 1 | 1 | 0.867 | 0.935 | 0.388 | 0.519 |
| 2500 | 1 | 1 | 1 | 1 | 0.929 | 0.972 | 0.465 | 0.613 |
| 3000 | 1 | 1 | 1 | 1 | 0.964 | 0.986 | 0.541 | 0.693 |
| 3500 | 1 | 1 | 1 | 1 | 0.982 | 0.995 | 0.604 | 0.759 |
| 4000 | 1 | 1 | 1 | 1 | 0.991 | 0.998 | 0.660 | 0.811 |
