# Supplementary material for: The Longitudinal Association Between Self‐esteem and Depressive Symptoms in Adolescents: Separating Between‐Person Effects from Within‐Person Effects
Source: Eur J Pers. 2018 Nov 5;32(6):653–71. doi: 10.1002/per.2179 (PMC6519152; doi:10.1002/per.2179)
Supplement: Supplementary file 2 — Open Practices Disclosure [file PER-32-653-s002.pdf]

OPEN PRACTICES DISCLOSURE

PLEASE COMPLETE AND RETURN TO PRODUCTION EDITOR AT PERPROOFS@WILEY.COM

***European Journal of Personality* manuscript #:**	**EJP-18-2551.R2 /** PER2179	**Corresponding author:**	**Maurits Masselink**

Articles accepted to *European Journal of Personality* are eligible to earn badges that recognize open scientific practices: publicly available data, material, or preregistered research plans. Please read more about the badges in the Open Science Framework wiki and FAQ.

☐ **Please check this box if you are not interested in participating.**

Even if you are not participating, please sign the form at the bottom of the next page before returning.

If you choose to participate, this form will be posted with your article as supplemental online material.

To apply for one or more badges acknowledging open practices, please check the appropriate box(es) below and provide the information requested in the relevant sections. You will not qualify for a badge for a given item unless you can provide a URL, doi, or other **permanent path** for accessing the specified information in a **public, open-access repository. Qualifying public, open-access repositories are committed to preserving data, materials, and/or registered analysis plans and keeping them publicly accessible via the web into perpetuity.** Examples include the Open Science Framework (OSF) and the various Dataverse networks. Hundreds of other qualifying data/materials repositories are listed at http://re3data.org/ and http://databib.org/. Preregistration of an analysis plan must take place via a publicly accessible registry system (e.g., OSF, ClinicalTrials.gov or other trial registries in the WHO Registry Network, institutional registration systems). **Personal websites and most departmental websites do not qualify as repositories.**

Authors who wish to publicly post third-party material in their data, materials, or preregistration plan must have the proper authority or permission agreement in order to do so.

☑ **Application for Open Data Badge**

1. Provide the URL, doi, or other **permanent path** for accessing the data in a **public, open-access repository**:

   https://osf.io/cxc6f/

   ☑ Confirm that there is sufficient information for an independent researcher to reproduce **all of the reported results**, including codebook if relevant.

☑ **Application for Open Materials Badge**

1. Provide the URL, doi, or other **permanent path** for accessing the materials in a **public, open-access repository**:

   https://osf.io/cxc6f/

   ☑ Confirm that there is sufficient information for an independent researcher to reproduce **all of the reported methodology**.

☑ **Application for Preregistration Badge**

1. Provide the URL, doi, or other **permanent path** to the registration in a **public, open-access repository**.*
   Study 3 was pre-registered: https://osf.io/y3zcn/

☑ **Application for Preregistered + Analysis Plan Badge**

(Application for this badge supersedes the above Preregistration Badge without an analysis plan)

1. Provide the URL, doi, or other **permanent path** to the registration in a **public, open-access repository.***
   https://osf.io/y3zcn/

2. Was the analysis plan registered prior to examination of the data or observing the outcomes? If no, explain.**
   Yes

3. Were there additional registrations for the study other than the one reported? If yes, provide links and explain.*
   no

4. Were there any changes to the preregistered analysis plan for the primary confirmatory analysis? If yes, explain.**
   Yes, we report the following in the manuscript:
   "The analyses for Study 3 were pre-registered (https://osf.io/y3zcn/). The analyses performed deviated from the pre-registration in a few respects. For transparency we explain how these changes came about. We initially planned to conduct confirmatory factor analyses on the measures, save the factor scores, and use the factor scores as input in the RI-CLPM. However, adding one-level measurement models to the RI-CLPM resulted in a model in which the within-person and between-person variance could no longer be accurately teased apart. We therefore decided against extending the RI-CLPM with a measurement model, but to rely on the common practice of using mean scores instead (in line with the earlier studies using RI-CLPM, e.g. by Hamaker et al., 2015 and Keijsers, 2016). Initially, factor scores were used in Study 1 and 2 as well, but for the abovementioned reason, we re-ran the analyses in Study 1 and 2 with mean scores instead of factor scores. Some outcomes differed somewhat depending on whether factor or mean scores were used, which explains the differences in the description of the results of Study 1 and 2 in the present article and the short references to these results in the pre-registration."

5. Are all of the analyses described in the registered plan reported in the article? If no, explain.*
   No, we explain above why we changed our analysis strategy.

*No badge will be awarded if (1) is not provided, **or** if (3) is answered "yes" without strong justification, **or** if (5) is answered "no" without strong justification.
**If the answer to (2) is "no," the notation DE (Data Exist) will be added to the badge, indicating that registration postdates realization of the outcomes but predates analysis. If the answer to (4) is "yes" with strong justification for changes, the notation TC (Transparent Changes) will be added to the badge, indicating that the analysis plan was altered but the preregistered analyses and rationale for the change are provided.

By signing below, authors affirm that the above information is accurate and complete, that any third-party material has been reproduced or otherwise made available only with the permission of the original author or copyright holder, and that publicly posted data do not contain information that would allow individuals to be identified without consent.

Name: **Maurits Masselink** **Date:** **23 Oct. 18**
